# Relationships among highly sensitive personality (HSP), neuroticism, mental health, neurodevelopmental traits, and adverse childhood experiences (ACEs)

**DOI:** 10.1002/pcn5.70322

**Published:** 2026-03-26

**Authors:** Akari Matsuzawa, Tsubasa Sasaki, Eiji Shimizu

**Affiliations:** ^1^ Department of Cognitive Behavioral Physiology, Graduate School of Medicine Chiba University Chiba Chiba Japan; ^2^ Research Center for Child Mental Development Chiba University Chiba Chiba Japan; ^3^ United Graduate School of Child Development The University of Osaka, Kanazawa University, Hamamatsu University School of Medicine, Chiba University and University of Fukui Suita Osaka Japan; ^4^ Cognitive Behavioral Therapy Center Chiba University Hospital Chiba Chiba Japan

**Keywords:** adverse childhood experiences, highly sensitive personality, mental health, neurodevelopmental traits, neuroticism

## Abstract

**Aim:**

Awareness of mental health is important to reduce stigma and discrimination and increase access to care. From the viewpoint of mental health conditions, this study examined the relationships among highly sensitive personality (HSP) traits and neuroticism in the general population.

**Methods:**

After obtaining informed consents, an anonymous web‐based survey for adult participants with equal data collection across genders and age groups (20s, 30s, and 40s) was conducted using self‐report questionnaires about HSP, neuroticism, mental health conditions including depression, anxiety, autism spectrum disorder (ASD), attention‐deficit/hyperactivity disorder (ADHD), trauma‐related stress, adverse childhood experiences (ACEs), and awareness of HSP and neurodevelopmental disorders. Participants were divided into the three groups based on HSP or neuroticism, with low, medium, and high groups defined by the mean value ± 1 SD.

**Results:**

Data from 2593 participants were analyzed. High‐HSP and high‐neuroticism groups exhibited higher levels of depression, anxiety, ASD, ADHD traits, trauma‐related stress, and ACEs, compared to the other medium and low groups, respectively. Regarding ACEs, both the high‐HSP and high‐neuroticism groups showed significantly high rates of psychological abuse and neglect. While the high‐neuroticism group showed broad associations across all ACE categories, the high‐HSP group showed specific and comparable, or slightly stronger, associations with household mental disorders and physical abuse. In the high‐HSP group, more participants answered that being HSP or having a neurodevelopmental disorder was part of their identity.

**Conclusion:**

High HSP and high neuroticism show mental health similarities, but HSP uniquely internalizes traits as identity.

## INTRODUCTION

### Highly sensitive personality

Highly sensitive personality (HSP) is a concept introduced to describe individuals with high “sensory‐processing sensitivity” (SPS), a trait estimated to be present in 15%–20% of the population.^1^ SPS leads to two different strategies when faced with new stimuli: either engaging with the stimuli exploratory or distancing oneself and becoming cautious.^2^ This trait reflects individual differences in environmental sensitivity and is considered adaptive in the evolutionary process of the central nervous system.^3^ Twin studies have reported that the heritability of environmental sensitivity is about 47%.^4^ Aron and Aron developed a self‐report questionnaire called the Highly Sensitive Person Scale, initially proposing a one‐factor structure.^1^ However, subsequent discussions regarding the factor structure have highlighted specific dimensions of sensitivity, most notably “low sensory threshold” (e.g., discomfort from loud noises or when many things are happening at once) and “ease of excitation” (e.g., being easily startled or influenced by others' moods).^5^ While the factor structure continues to be debated, significant scientific criticism regarding validity and insufficient validation makes it challenging to consider HSP a scientifically established personality trait.

Among adults, HSP has been reported to show a positive correlation with neuroticism and openness, whereas in children, HSP is positively correlated only with neuroticism.^6^ Furthermore, individuals with higher HSP tendencies are more likely to experience depressive and anxiety symptoms.^7^ In recent years, the term “HSP” has been increasingly heard from patients and those around them in outpatient and counseling settings. This study reports insights gained regarding the characteristics of individuals referred to as HSP.

### Neuroticism

Neuroticism, characterized by tendencies such as being anxious, easily distressed, and emotionally unstable, is a personality trait that is prone to experiencing negative emotions. It is one of the five major dimensions of personality traits in the Big Five personality model, which is widely recognized worldwide. The other four dimensions are extraversion, openness, conscientiousness (or industriousness), and agreeableness. Neuroticism is related to symptoms of depression, anxiety disorders, and psychiatric diagnoses, as demonstrated by a meta‐analysis of 59 longitudinal studies with a total of 443,313 participants,^8^ along with numerous other studies.^9^, ^10^, ^11^ Furthermore, the strength of the association between childhood trauma and neuroticism has also been noted.^12^ Twin studies have reported heritability rates of 48% for neuroticism, 54% for extraversion, 57% for openness, 49% for conscientiousness (industriousness), and 42% for agreeableness.^13^

### Neurodevelopmental traits, HSP, and neuroticism

The relationship between high‐HSP tendencies, such as being uncomfortable with loud noises or many things happening at once, and sensory hypersensitivity or sensory hypo‐responsiveness traits in ASD has been suggested, as well as the relationship with ADHD.^14^ On the other hand, a study using the self‐report questionnaire AQ‐50 for ASD traits found that higher ASD traits were associated with higher neuroticism, lower extraversion, and lower conscientiousness.^15^ In adults with ADHD, the Big Five personality traits showed that inattention symptoms positively correlated with neuroticism but negatively correlated with conscientiousness. Furthermore, hyperactivity and impulsivity symptoms were related to extraversion and agreeableness.^16^ Additionally, in adults with ADHD, the neurocognitive profile was related to openness, and ADHD patients showed higher neuroticism, lower extraversion, lower conscientiousness, and lower agreeableness compared to healthy controls.^17^ ASD and ADHD, as well as the co‐occurrence of ASD and ADHD, are known to frequently involve comorbid mood disorders, including anxiety and depression.^18^ Furthermore, the comorbidity of anxiety and depression has been reported in ADHD.^19^ Therefore, the association between neurodevelopmental traits in ASD, ADHD, and high neuroticism may be due to comorbid depression and anxiety. However, to date, no comprehensive studies have examined the neurodevelopmental traits, HSP, Big Five personality traits, and neuroticism about depression and anxiety symptoms.

### Adverse childhood experiences, HSP, and neuroticism

Adverse childhood experiences (ACEs) have been reported to show a positive correlation with neuroticism and a negative correlation with conscientiousness.^20^ However, to date, no studies have examined the relationship between HSP and ACEs. Exposure to ACEs has been shown in multiple studies to increase the risk of cognitive, social, emotional, and health‐related problems in both children and adults.^21^, ^22^ Nevertheless, whether individuals develop such problems under adverse circumstances varies. In other words, sensitivity to the same environment differs from person to person.

### The growing social interest in HSP has led to accompanying issues

HSP attracts attention because of not only negative traits such as low sensory threshold and ease of excitation but also positive traits like aesthetic sensitivity. In many countries, including Japan, HSP is widely discussed in social media, books, and television, and some individuals publicly identify as HSPs online. A study of Swedish online forums and blogs suggested that identifying as HSP may promote self‐understanding, identity formation, and community building.^23^ However, because HSP is neither scientifically established nor a psychiatric diagnosis, increased public attention has led some individuals with mental disorders to adopt “HSP” as part of their identity, raising clinical concerns.

Ioannou et al. (2023) used the cultural formulation interview (CFI) to examine how individuals identifying as HSPs employ the concept as a cultural explanatory model of distress rather than as a diagnosis.^24^ Since HSP is absent from diagnostic systems such as DSM‐5, the CFI offers a suitable framework for clarifying culturally shaped interpretations of suffering. Within this framework,^25^ HSP can be conceptualized as a cultural concept of distress (CCD), whereby symptoms are reframed as expressions of “innate sensitivity,” potentially reducing stigma and supporting coherent self‐understanding. Here, “cultural distress” refers to culturally shaped modes of experiencing and expressing suffering, helping explain why individuals with high‐HSP traits may interpret their distress or trauma responses through the lens of HSP.

Given these issues, psychiatric research is needed to examine how identification with HSP forms part of self‐identity, how HSP relates to depression, anxiety, neurodevelopmental traits, and trauma, and whether these associations resemble those observed for neuroticism, a well‐established personality trait.

## AIM

Based on the hypothesis that having HSP is nearly identical to having high neuroticism, this study investigates the associations between HSP and several constructs commonly examined in psychiatry and known to be related to neuroticism, namely, depressive symptoms, anxiety symptoms, neurodevelopmental traits, personality traits, ACEs, and traumatic stress. Furthermore, by comparing these associations with those observed for neuroticism, the study aims to gain insights into the nature of what it means to be an HSP.

## METHODS

### Participants

After approval from the Ethics Review Committee of the Graduate School of Medicine, Chiba University (Approval Number: M10716), a research description document was made publicly available online, and adult participants who provided informed consent were surveyed. The survey was conducted anonymously online through the consumer monitor provider Freeasy (iBRIDGE Corp., Tokyo, Japan). As compensation, participants received points equivalent to 1 Japanese Yen per question. The survey period was in August 2024, with equal data collection across genders and age groups (20s, 30s, and 40s) (ranging from 20 to 49 years old). Data collection was concluded when online survey responses were obtained from 3000 adult participants.

After excluding individuals with invalid responses on the two Lie scale questions (“Is 1 plus 2 equal to 2?” and “Is 7 minus 2 equal to 5?”), 2593 participants (1257 men and 1336 women) were included in the final analysis. HSP traits were measured using the 19‐item Japanese version of the Highly Sensitive Person Scale, initially developed by Aron and Aron (1997). This scale assesses SPS, with each item rated on a 5‐point Likert scale. Higher total scores indicate greater sensitivity. Neuroticism was assessed using the Ten Item Personality Inventory – Japanese version (TIPI‐J). Neuroticism scores were calculated as the mean of two items (with one reverse‐coded).

### Assessment scales

(i) Highly Sensitive Person Scale Japanese version 19 items (HSPS‐J19)

Aron and Aron developed a self‐report questionnaire with 27 items using a 7‐point scale, designed to assess positive and negative cognitive and emotional responses to various environmental stimuli.^1^ This achievement allowed for the evaluation of individuals' environmental sensitivity. In Japan, the self‐report questionnaire was translated into Japanese, creating the 19‐item HSPS‐J19.^26^ The minimum score is 19, and the maximum score is 133. The HSP scale was originally developed as a one‐factor structure. Still, in recent years, a three‐factor structure including low sensory threshold, ease of excitation, and aesthetic sensitivity has become more widely accepted.^27^, ^28^

(ii) Ten Item Personality Inventory – Japanese version

The TIPI‐J is the Japanese version of a shortened self‐report questionnaire designed to measure the Big Five personality traits (neuroticism, extraversion, agreeableness, conscientiousness, and openness).^29^ Each trait is measured with two items, one of which is a reversed item. The questionnaire consists of 10 items on a 7‐point scale. For neuroticism, the two items are: “I tend to worry and get upset” and “I think I am calm and stable (reverse‐scored item).”

(iii) Patient Health Questionnaire‐9 (PHQ‐9)

The PHQ‐9 is a self‐report questionnaire used to assess depressive symptoms, consisting of nine questions. In this study, the Japanese version is used.^30^ It is scored on a 4‐point scale ranging from 0 to 3 points. The severity levels are as follows: 0–4 points is minimal, 5–9 points is mild, 10–14 points is moderate, 15–19 points is moderately severe, and 20–27 points is severe. A cutoff score of 10 points is used.

(iv) Generalized Anxiety Disorder‐7 (GAD‐7)

The GAD‐7 is a self‐report questionnaire used to assess anxiety, consisting of seven questions. In this study, the Japanese version is used.^31^ It is scored on a 4‐point scale ranging from 0 to 3 points. The severity levels are as follows: 0–4 points is minimal, 5–9 points is mild, 10–14 points is moderate, and 15–21 points is severe. A cutoff score of 10 points is used.

(v) Adult ADHD Self‐Report Scale ver1.1 (ASRSv1.1)

The ASRSv1.1 is the World Health Organization's self‐report scale for adult ADHD, consisting of 18 items. It uses a 5‐point scale ranging from 0 to 4 points. The first six items make up Part A, and the remaining 12 items make up Part B. Part A is used for screening, and if four or more items score 2 or 3 points, the likelihood of ADHD is considered very high. In this study, the Japanese version is used. The total score for Part A and Part B ranges from 0 to 72 points, with a cutoff value of 24 or more.^32^, ^33^

(vi) Autism‐Spectrum Quotient test‐50 (AQ‐50)

The AQ‐50 is a self‐report questionnaire used to assess autism spectrum traits, using a 4‐point scale. The minimum score is 0, and the maximum score is 50. In this study, the Japanese version is used.^34^ The cutoff value is 33 or more.^35^

(vii) Impact of Event Scale‐Revised (IES‐R)

The IES‐R is a 22‐item self‐report scale assessing intrusion, avoidance, and hyperarousal symptoms, rated on a 5‐point scale. In this study, the Japanese version was used.^36^ Total scores range from 0 to 88, with a cutoff score of 24.^37^ Although originally developed for DSM‐based Criterion A traumatic events, the IES‐R has also been applied to highly distressing but non–life‐threatening stressors, including the assessment of stress‐related symptoms in patients with treatment‐refractory depression^38^ and in studies examining its psychometric properties among victims of workplace bullying.^39^

In this study, “events involving strong stress” included not only life‐threatening traumatic events but also a broader range of stressful experiences. Participants selected applicable events from ten predefined categories, such as past abuse or neglect in the home, interpersonal difficulties within the home, school bullying, academic or career‐related problems, work‐related issues not involving interpersonal conflict, and other significant adverse events.

(viii) Adverse childhood experiences

The ACEs is a self‐report questionnaire used to assess the adversity experienced during childhood. These cover the possible forms of maltreatment (physical, psychological, and sexual abuse, and physical and psychological neglect) and household dysfunction (parental separation/divorce, household physical violence, household substance abuse, household mental disorders, incarcerated household member).^40^ It consists of 10 items with a 2‐point scale, asking whether the experience occurred or not (“Yes” or “No”).^41^ In this study, the Japanese version is used.

### Statistical analysis

To examine the specific characteristics of individuals with high trait levels and to identify potential “threshold effects” regarding mental health risks, we adopted a trichotomous classification approach rather than treating the scales as continuous variables. Theoretically, high sensitivity is estimated to be present in approximately 15%–20% of the population.^1^ In a normal distribution, the area above +1 SD corresponds to approximately 15.9%, providing a statistical criterion that aligns well with the theoretical prevalence of HSP.

Therefore, following the method used by Ueno et al.,^42^ participants were classified into three groups (low HSP, medium HSP, and high HSP) based on the total HSPS J‐19 score, using the mean ± 1 SD (*M* = 78.13, SD = 19.953) as the cutoff criteria. Unlike a median split or simple dichotomy, this method isolates the “medium” group (within ±1 SD), preventing the aggregation of average scores with the extremes and allowing for a more sensitive comparison of the high‐trait group against the others. Neuroticism was also classified into three groups (low, medium, and high) using the same criterion (mean ± 1 SD) to ensure comparability between the high‐scoring layers of both traits.

For each psychological measure, the normality was assessed using the Shapiro–Wilk test. The Kruskal–Wallis test and post hoc multiple comparison methods were used to compare HSP and neuroticism across the three groups, as normality was not observed in the psychological scales.　For “participant background,” “the most stressful event experienced in the past,” “each item of ACEs,” and “recognition of HSP and developmental disorders,” chi‐square tests were conducted, and residual analysis was performed for group comparisons.　The statistical analysis was conducted using spss Version 29.0 (IBM).

## RESULTS

Out of the 3000 adult participants, 2593 individuals (1257 men and 1336 women) were included in the analysis after excluding those who answered the Lie scale questions incorrectly. Following the method of Ueno et al.,^42^ participants were classified into three groups (low‐HSP group, medium‐HSP group, high‐HSP group) based on their total HSPS‐J19 score, using the mean ± 1 SD (*M* = 78.13, SD = 19.953). The low‐HSP group comprised 354 participants (13.7%, *M* = 43.03, SD = 12.345), the medium‐HSP group comprised 1876 participants (72.3%, *M* = 78.89, SD = 9.921), and the high‐HSP group comprised 363 participants (14.0%, *M* = 108.45, SD = 7.933). Demographic information for each group is shown in Table 1. In the high‐HSP group, there were 254 women (representing 19.0% of the total 1336 female participants) and 109 men (8.7% of the total 1257 male participants). A chi‐square test revealed that women were significantly more prevalent than men (*p* < 0.001). Regarding age distribution, the proportions of participants in their 20s, 30s, and 40s were as follows: 120 individuals in their 20s (14.6% of the total 822 participants in this age group), 131 in their 30s (15.1% of the total 870), and 112 in their 40s (12.4% of the total 901). A chi‐square test showed no significant differences across age groups (*p* = 0.521). For comparison, participants were also classified into three groups based on their neuroticism scores on the TIPI‐J, using the mean ± 1 SD (*M* = 8.48, SD = 2.538). The low neuroticism group comprised 286 participants (11.0%, *M* = 3.97, SD = 1.082), the medium neuroticism group comprised 1977 participants (*M* = 8.39, SD = 1.400), and the high‐neuroticism group comprised 330 participants (*M* = 12.88, SD = 0.846). Demographic information for each group is shown in Table 2.

**Table 1:** Sociodemographic characteristics of the participants by HSP group (*n* = 2593).

Variable	Category	Total (100%)	%	Low‐HSP *n* = 354 (13.7%)	%	Medium‐HSP *n* = 1876 (72.3%)	%	High‐HSP *n* = 363 (14.0%)	%	
Gender	Female	1336	100.0	150	11.2	932	69.8	254	19.0	
	Male	1257	100.0	204	16.2	944	75.1	109	8.7	Total: 2593
Age	20s	822	100.0	115	14.0	587	71.4	120	14.6	
	30s	870	100.0	113	13.0	626	72.0	131	15.1	
	40s	901	100.0	126	14.0	663	73.6	112	12.4	Total: 2593

Abbreviation: HSP, highly sensitive personality.

**Table 2:** Sociodemographic characteristics of the participants by neuroticism group (*n* = 2593).

Variable	Category	Total (100%)	%	Low‐neuroticism *n* = 286 (11.0%)	%	Medium‐neuroticism *n* = 1977 (76.2%)	%	High‐neuroticism *n* = 330 (12.7%)	%	
Gender	Female	1336	100.0	132	9.9	987	73.9	217	16.2	
	Male	1257	100.0	154	12.3	990	78.8	113	9.0	Total: 2593
Age	20s	822	100.0	89	10.8	634	77.1	99	12.0	
	30s	870	100.0	81	9.3	666	76.6	123	14.1	
	40s	901	100.0	116	12.9	677	75.1	108	12.0	Total: 2593

Both the high‐HSP group and the high‐neuroticism group had a higher proportion of women. In the high‐neuroticism group, there were 217 women (16.2% of the total 1336 female participants) and 113 men (9.0% of the total 1257 male participants), with a chi‐square test showing that women were significantly more prevalent (*p* < 0.001). Age distribution in this group was as follows: 99 individuals in their 20s (12.0% of the total 822 participants in this age group), 123 in their 30s (14.1% of the total 870), and 108 in their 40s (12.0% of the total 901). A chi‐square test revealed no significant differences across age groups (*p* = 0.115).　Background information regarding psychiatric history, excluding gender and age, is shown in Tables 3 and 4. Of the 2593 participants, 669 (25.8%) reported a history of psychiatric outpatient visits. Self‐reported rates of psychiatric diagnoses were as follows: depression, 13.8%; anxiety disorder, 10.5%; and developmental disorder, 6.1%. The high‐HSP group had significantly higher rates of psychiatric outpatient history, current psychiatric visits, psychiatric diagnoses, and diagnoses of depression, anxiety disorder, developmental disorder, ASD, and ADHD compared with the medium and low‐HSP groups (Table 3). Similarly, the high‐neuroticism group showed significantly higher rates for these variables compared with the other two neuroticism groups (Table 4).

**Table 3:** Participant background characteristics by HSP group (*n* = 2593).

Category	Total	%	Low‐HSP *n* = 354 (13.7%)		AR	Medium‐HSP *n* = 1876 (72.3%)		AR	High‐HSP *n* = 363 (14.0%)		AR	*p*	*V*
			*n*	%		*n*	%		*n*	%			
History of psychiatric outpatient treatment	669	25.8	30	8.5	−8	445	23.7	−3.9	194	53.4	13	<0.001	0.281
Currently undergoing psychiatric treatment	345	13.3	12	3.4	−5.9	218	11.6	−4.1	115	31.7	11.1	<0.001	0.233
History of psychiatric diagnosis	496	19.1	15	4.2	−7.7	312	16.6	−5.2	169	46.6	14.3	<0.001	0.301
History of depression diagnosis	359	13.8	16	4.5	−5.5	228	12.2	−4	115	31.7	10.6	<0.001	0.221
History of anxiety disorder diagnosis	273	10.5	17	4.8	−3.8	160	8.5	−5.4	96	26.4	10.7	<0.001	0.213
History of developmental disorder diagnosis	157	6.1	11	3.1	−2.5	89	4.7	−4.5	57	15.7	8.3	<0.001	0.165
History of ASD diagnosis	120	4.6	8	2.3	−2.3	72	3.8	−3.1	40	11.0	6.3	<0.001	0.125
History of ADHD diagnosis	102	3.9	7	2.0	−2	54	2.9	−4.5	41	11.3	7.8	<0.001	0.154

*Note*: The percentage for total represents the proportion relative to the total number of people, while the percentage for each group represents the proportion relative to the number of people in that specific group.

Abbreviations: ADHD, attention‐deficit/hyperactivity disorder; AR, adjusted residual; ASD, autism spectrum disorder; HSP, highly sensitive personality.

**Table 4:** Participant background characteristics by neuroticism group (*n* = 2593).

Category	Total	%	Low‐neuroticism *n* = 286 (11.0%)			Medium‐neuroticism *n* = 1977 (76.2%)			High‐neuroticism *n* = 330 (12.7%)			*p*	*V*
			*n*	%	AR	*n*	%	AR	*n*	%	AR		
History of psychiatric outpatient treatment	669	25.8	36	12.6	−5.4	464	23.5	−4.9	169	51.2	11.3	<0.001	0.235
Currently undergoing psychiatric treatment	345	13.3	14	4.9	−4.4	227	11.5	−4.9	104	31.5	10.4	<0.01	0.213
History of psychiatric diagnosis	496	19.1	26	9.1	−4.6	340	17.2	−4.5	130	39.4	10	<0.01	0.207
History of depression diagnosis	359	13.8	15	5.2	−4.5	247	12.5	−3.6	97	29.4	8.8	<0.01	0.184
History of anxiety disorder diagnosis	273	10.5	16	5.6	−2.9	181	9.2	−4.1	76	23.0	7.9	<0.01	0.16
History of developmental disorder diagnosis	157	6.1	11	3.8	−1.7	105	5.3	−2.8	41	12.4	5.2	<0.01	0.104
History of ASD diagnosis	120	4.6	12	4.2	−0.4	78	3.9	−3	30	9.1	4.1	<0.001	0.081
History of ADHD diagnosis	102	3.9	7	2.4	−1.4	64	3.2	−3.3	31	9.4	5.5	<0.01	0.108

*Note*: The percentage for total represents the proportion relative to the total number of people, while the percentage for each group represents the proportion relative to the number of people in that specific group.

Abbreviations: ADHD, attention‐deficit/hyperactivity disorder; AR, adjusted residual; ASD, autism spectrum disorder.

Positive correlations were observed between HSPS‐J19 scores and several psychological scales. The six strongest correlations were with GAD‐7 (*ρ* = 0.533), PHQ‐9 (*ρ* = 0.507), ASRS (*ρ* = 0.490), neuroticism (*ρ* = 0.468), IES‐R (*ρ* = 0.367), and AQ (*ρ* = 0.300) (data not shown). For each psychological scale, including measures of depression, anxiety, and developmental traits, group comparisons were conducted among the three HSP groups and among the three neuroticism groups using the Kruskal–Wallis test with post hoc comparisons. The results are presented in Tables 5 and 6. The high‐HSP group scored significantly higher than the medium and low‐HSP groups on PHQ‐9, GAD‐7, ASRS, AQ‐50, TIPI‐J neuroticism, IES‐R, and ACEs, while scoring significantly lower on TIPI‐J extraversion. Notably, the mean PHQ‐9 score in the high‐HSP group exceeded the cutoff for moderate depression (≥10), indicating clinically significant depression. Likewise, their mean GAD‐7 score exceeded the moderate anxiety cutoff (≥10), their mean ASRS score exceeded the ADHD cutoff (≥24), and their mean IES‐R score exceeded the PTSD cutoff (≥24) (Table 5).

**Table 5:** Psychological scale scores by HSP group (*n* = 2593).

Variable	Category	Low‐HSP *n* = 354 (13.7%)			Medium‐HSP *n* = 1876 (72.3%)			High‐HSP *n* = 363 (14.0%)			Kruskal–Wallis	Tukey‐HSD
		Range	Median (IQR)	Mean (SD)	Range	Median (IQR)	Mean (SD)	Range	Median (IQR)	Mean (SD)		
Psychological scale	HSPSJ‐19	19–58	45 (34–54)	43.03 (12.345)	59–98	78 (71–86)	78.89 (9.921)	99–133	107 (102–113)	108.45 (7.933)		
	LST	7–31	14 (10–18)	14.32 (5.258)	10–49	29 (26–33)	29.56 (5.484)	32–49	43 (40–46)	42.65 (3.970)	a**, b**, c**	a**, b**, c**
	EOE	8–30	17 (11–20)	16.15 (5.703)	15–53	33 (29–37)	33.08 (5.882)	29–56	46 (43–50)	46.71 (4.784)	a**, b**, c**	a**, b**, c**
	AES	4–28	12 (8–17)	12.57 (5.798)	4–28	16 (14–18)	16.25 (3.436)	4–28	19 (16–22)	19.08 (4.378)	a**, b**, c**	a**, b**, c**
	PHQ‐9	0–27	1 (0–3)	2.47 (4.108)	0–27	6 (2–11)	7.23 (6.009)	0–27	14 (8–19)	13.79 (7.323)	a**, b**, c**	a**, b**, c**
	GAD‐7	0–21	0 (0–1)	1.62 (3.440)	0–21	4 (1–8)	4.93 (4.821)	0–21	11 (7–15)	11 (5.825)	a**, b**, c**	a**, b**, c**
	ASRS	0–71	1 (0–9)	6.5 (10.262)	0–72	17 (6–26)	17.34 (13.44)	0–72	31 (20–41)	31.70 (16.665)	a**, b**, c**	a**, b**, c**
	AQ‐50	4–39	23 (17–26)	21.05 (6.511)	4–44	25 (21–28)	23.92 (6.195)	7–45	27 (23–31.5)	27.23 (6.647)	a**, b**, c**	a**, b**, c**
	TIPI‐J											
	Neuroticism	2–14	7 (5–8)	6.62 (2.378)	2–14	8 (7–10)	8.44 (2.262)	2–14	11 (8–13)	10.5 (2.57)	a**, b**, c**	a**, b**, c**
	Extraversion	2–14	8 (6–10)	8.09 (2.86)	2–14	7 (5–8)	6.85 (2.573)	2–14	6 (4–8)	5.88 (2.806)	a′**, b′**, c′**	a′**, b′**, c′**
	Agreeableness	2–14	9 (8–11)	9.05 (2.321)	2–14	9 (8–11)	9.29 (2.11)	2–14	9 (8–10)	8.94 (2.372)	a′*	a′*
	Conscientiousness	2–14	8 (8–10)	8.81 (2.415)	2–14	8 (6–9)	7.55 (2.506)	2–14	8 (5–9)	7.22 (3.01)	b′**, c′**	b′**, c′**
	Openness	2–14	8 (7–9)	7.91 (2.437)	2–14	8 (6–8)	7.17 (2.280)	2–14	8 (5–9)	7.23 (2.815)	b′**, c′**	b′**, c′**
	IES‐R	0–88	8 (0–26.75)	15.18 (17.689)	0–88	23 (7–42)	25.34 (19.004)	0–88	46 (27–60)	43.19 (22.745)	a**, b**, c**	a**, b**, c**
	Intrusion	0–32	2 (0–10)	5.42 (6.624)	0–32	8 (2–15)	8.96 (7.294)	0–32	17 (9–22)	15.80 (9.139)	a**, b**, c**	a**, b**, c**
	Avoidance	0–32	3 (0–10)	5.82 (6.830)	0–32	9 (3–16)	9.40 (7.103)	0–32	16 (10–21)	15.26 (8.034)	a**, b**, c**	a**, b**, c**
	Hyperarousal	0–24	1 (0–6)	3.67 (4.967)	0–24	6 (1–11)	6.37 (5.568)	0–24	12 (6–17)	11.75 (7.057)	a**, b**, c**	a**, b**, c**
	ACEs	0–10	0 (0–3)	2.12 (3.359)	0–10	1 (0–4)	2.34 (3.198)	0–10	2 (0–5)	3.13 (3.179)	a**, b**, c*	a**, b**

*Note*: **p < 0.01; *p＜0.05; a, high‐HSP was higher than medium‐HSP; b, high‐HSP was higher than low‐HSP; c, medium‐HSP was higher than low‐HSP; a′, high‐HSP was **lower** than medium‐HSP; b′, high‐HSP was **lower** than low‐HSP; c′, medium‐HSP was **lower** than low‐HSP; n.s., not significant.

Abbreviations: ACE, adverse childhood experience; AES, aesthetic sensitivity; AQ‐50, Autism‐Spectrum Quotient test‐50; ASRS, Adult attention‐deficit/hyperactivity disorder Self‐Report Scale; EOE, ease of excitation; GAD‐7, Generalized Anxiety Disorder‐7; HSP, highly sensitive personality; HSPSJ‐19, Highly Sensitive Person Scale Japanese version 19 items; IES‐R, Impact of Event Scale‐Revised; IQR, interquartile range; LST, low sensory threshold; PHQ‐9, Patient Health Questionnaire‐9; TIPI‐J, Ten Item Personality Inventory – Japanese version; Tukey‐HSD, Tukey's honestly dignificant difference.

**Table 6:** Psychological scale scores by neuroticism group (*n* = 2593).

Variable	Category	Low‐neuroticism *n* = 286 (11.0%)			Medium‐neuroticism *n* = 1977 (76.2%)			High‐neuroticism *n* = 330 (12.7%)			Kruskal–Wallis	Tukey‐HSD
		Range	Median (IQR)	Mean (SD)	Range	Median (IQR)	Mean (SD)	Range	Median (IQR)	Mean (SD)		
Psychological scale	HSPSJ‐19	19–113	65 (51–76)	63.49 (19.605)	19–133	78 (69–88)	76.58 (18.5)	25–129	96 (86–108)	95.24 (16.275)	a**, b**, c**	a**, b**, c**
	LST	7–46	23 (16–29.75)	22.84 (9.226)	7–49	29 (25–34)	28.9 (8.297)	7–49	38 (33–44)	37.38 (8.193)	a**, b**, c**	a**, b**, c**
	EOE	8–56	24 (16–29)	23.3 (9.514)	8–56	32 (28–38)	32.37 (8.705)	8–56	43 (39–48)	42.62 (7.738)	a**, b**, c**	a**, b**, c**
	AES	4–28	18 (15–21)	17.35 (5.074)	4–28	16 (14–19)	16.12 (4.14)	4–26	16 (13–18)	15.24 (4.512)	a**, b**, c**	a**, b**, c**
	PHQ‐9	0–26	2 (0–4)	3.51 (4.942)	0–27	6 (2–11)	7.16 (6.377)	0–27	13 (8–17)	12.95 (6.664)	a**, b**, c**	a**, b**, c**
	GAD‐7	0–20	0 (0–2)	1.95 (3.678)	0–21	4 (0–8)	5.00 (5.087)	0–21	10 (6–14.75)	10.25 (5.628)	a**, b**, c**	a**, b**, c**
	ASRS	0–72	6 (0–15)	10.07 (11.98)	0–72	16 (4–27)	17.36 (14.739)	0–72	26 (18–36)	27.65 (14.839)	a**, b**, c**	a**, b**, c**
	AQ‐50	4–39	19 (13–23.75)	18.41 (6.648)	4–44	25 (21–27)	24.06 (5.891)	9–45	28 (24.25–33)	28.42 (6.342)	a**, b**, c**	a**, b**, c**
	TIPI‐J											
	Neuroticism	2–5	4 (3–5)	3.97 (1.082)	6–11	8 (8–9)	8.39 (1.4)	2–14	13 (12–14)	12.88 (0.846)		
	Extraversion	2–14	8 (5–11)	8.02 (3.433)	2–14	7 (5–8)	6.97 (2.463)	2–14	5 (3–7)	5.41 (2.810)	a′**, b′**, c′**	a′**, b′**, c′**
	Agreeableness	2–14	10 (8–11)	9.70 (2.234)	2–14	9 (8–11)	9.11 (2.089)	2–14	9 (8–11)	9.33 (2.582)	c′**	c′**
	Conscientiousness	2–14	10 (7.25–12)	9.31 (3.034)	2–14	8 (6–9)	7.74 (2.212)	2–14	5 (3–8)	5.82 (3.249)	a′**, b′**, c′**	a′**, b′**, c′**
	Openness	2–14	8 (6–10)	8.15 (2.968)	2–14	8 (6–8)	7.38 (2.124)	2–14	6 (4–8)	5.95 (2.827)	a′**, b′**, c′**	a′**, b′**, c′**
	IES‐R	0–88	7.5 (1–23)	15.36 (18.162)	0–88	24 (7–43)	25.96 (19.847)	0–88	40 (22.25–56)	39.02 (22.313)	a**, b**, c**	a**, b**, c**
	Intrusion	0–32	2 (0–8)	5.07 (6.705)	0–32	9 (2–16)	9.27 (7.564)	0–32	14 (7–21)	14.21 (9.133)	a**, b**, c**	a**, b**, c**
	Avoidance	0–32	3.5 (0–10.75)	6.22 (7.244)	0–32	9 (3–16)	9.6 (7.375)	0–32	14 (8–20)	13.56 (7.855)	a**, b**, c**	a**, b**, c**
	Hyperarousal	0–24	1 (0–5)	3.42 (5.015)	0–24	6 (1–11)	6.59 (5.737)	0–24	10 (5–16.75)	10.66 (7.203)	a**, b**, c**	a**, b**, c**
	ACEs	0–10	0 (0–2)	1.45 (2.615)	0–10	1 (0–5)	2.58 (3.406)	0–10	2 (0–4)	2.27 (2.361)	b**, c**	b**, c**

*Note*: **p < 0.01; *p < 0.05; a, high‐neuroticism was **higher** than medium‐neuroticism; b, high‐neuroticism was **higher** than low‐neuroticism; c, medium‐neuroticism was **higher** than low‐neuroticism; a′, high‐neuroticism was **lower** than medium‐neuroticism; b′, high‐neuroticism was **lower** than low‐neuroticism; c′, medium‐neuroticism was **lower** than low‐neuroticism; n.s., not significant.

Abbreviations: ACE, adverse childhood experience; AES, aesthetic sensitivity; AQ‐50, Autism‐Spectrum Quotient test‐50; ASRS, Adult attention‐deficit/hyperactivity disorder Self‐Report Scale; EOE, ease of excitation; GAD‐7, Generalized Anxiety Disorder‐7; HSPSJ‐19, Highly Sensitive Person Scale Japanese version 19 items; IES‐R, Impact of Event Scale‐Revised; IQR, interquartile range; LST, low sensory threshold; PHQ‐9, Patient Health Questionnaire‐9; TIPI‐J, Ten Item Personality Inventory – Japanese version; Tukey‐HSD, Tukey's honestly significant difference.

The high‐neuroticism group scored significantly higher than the medium and low neuroticism groups on HSPS‐J19, low sensory threshold, ease of excitation, aesthetic sensitivity, PHQ‐9, GAD‐7, ASRS, AQ‐50, and IES‐R. Conversely, TIPI‐J extraversion, conscientiousness, and openness scores were significantly lower. Their mean PHQ‐9, GAD‐7, ASRS, and IES‐R scores also exceeded the respective cutoffs for moderate depression, moderate anxiety, ADHD, and PTSD (Table 6). The results regarding the most stressful past events are shown in Tables 7 and 8. Among the participants, 123 (4.7%) identified “abuse/neglect at home” as the most stressful past event, 8.4% identified “interpersonal relationships at home (excluding abuse/neglect),” and 14.7% identified “bullying at school.”

**Table 7:** The most stressful event experienced in the past by HSP group (*n* = 2593).

Category	Total	%	Low‐HSP *n* = 354 (13.7%)			Medium‐HSP *n* = 1876 (72.3%)			High‐HSP *n* = 363 (14.0%)		
			*n*	%	AR	*n*	%	AR	*n*	%	AR
Past abuse/neglect in the family	123	4.7	12	3.4	−1.3	75	4.0	−2.9	36	9.9	5
Past relationships in the family (excluding abuse/neglect)	217	8.4	20	5.6	−2	154	8.2	−0.5	43	11.8	2.6
Past bullying at school	382	14.7	28	7.9	−3.9	271	14.4	−0.7	83	22.9	4.7
Past relationships at school (excluding bullying)	373	14.4	48	13.6	−0.5	281	15.0	1.4	44	12.1	−1.3
Past bullying/harassment at work	204	7.9	19	5.4	−1.9	152	8.1	0.7	33	9.1	0.9
Past relationships at work (excluding bullying/harassment)	318	12.3	43	12.1	−0.1	231	12.3	0.1	44	12.1	−0.1
Past non‐relationship‐related troubles: financial difficulties, childcare, children's education, caregiving	132	5.1	13	3.7	−1.3	102	5.4	1.3	17	4.7	−0.4
Past non‐relationship‐related troubles: academic issues or career path concerns	108	4.2	15	4.2	0.1	85	4.5	1.5	8	2.2	−2
Past non‐relationship‐related troubles: work or employment issues	162	6.2	30	8.5	1.9	118	6.3	0.1	14	3.9	−2
Other past events: disasters, incidents, accidents, illnesses, etc.	574	22.1	126	35.6	6.6	407	21.7	−0.9	41	11.3	−5.4
						*p* < 0.001	*v*＝0.156		

*Note*: The percentage for total represents the proportion relative to the total number of people, while the percentage for each group represents the proportion relative to the number of people in that specific group.

Abbreviations: AR, adjusted residual; HSP, highly sensitive personality.

**Table 8:** The most stressful event experienced in the past by neuroticism group (*n* = 2593).

Category	Total	%	Low‐neuroticism *n* = 286 (11.0%)			Medium‐neuroticism *n* = 1977 (76.2%)			High‐neuroticism *n* = 330 (12.7%)		
			*n*	%	AR	*n*	%	AR	*n*	%	AR
Past abuse/neglect in the family	123	4.7	15	5.2	0.4	80	4.0	−3	28	8.5	3.4
Past relationships in the family (excluding abuse/neglect)	217	8.4	20	7.0	−0.9	159	8.0	−1.1	38	11.5	2.2
Past bullying at school	382	14.7	35	12.2	−1.3	275	13.9	−2.1	72	21.8	3.9
Past relationships at school (excluding bullying)	373	14.4	38	13.3	−0.6	288	14.6	0.5	47	14.2	−0.1
Past bullying/harassment at work	204	7.9	17	5.9	−1.3	160	8.1	0.8	27	8.2	0.2
Past relationships at work (excluding bullying/harassment)	318	12.3	30	10.5	−1	249	12.6	0.9	39	11.8	−0.3
Past non‐relationship‐related troubles: financial difficulties, childcare, children's education, caregiving	132	5.1	13	4.5	−0.4	102	5.2	0.3	17	5.2	0.1
Past non‐relationship‐related troubles: academic issues or career path concerns	108	4.2	16	5.6	1.3	87	4.4	1.1	5	1.5	−2.6
Past non‐relationship‐related troubles: work or employment issues	162	6.2	26	9.1	2.1	117	5.9	−1.2	19	5.8	−0.4
Other past events: disasters, incidents, accidents, illnesses, etc.	574	22.1	76	26.6	1.9	460	23.3	2.5	38	11.5	−5
						*p* < 0.001	*v* = 0.112			

*Note*: The percentage for total represents the proportion relative to the total number of people, while the percentage for each group represents the proportion relative to the number of people in that specific group.

Abbreviation: AR, adjusted residual.

The high‐HSP group reported significantly higher rates of “abuse/neglect at home,” “interpersonal relationships at home (excluding abuse/neglect),” and “bullying at school” compared with the other HSP groups, while reporting significantly lower rates of “academic/career troubles unrelated to interpersonal relationships,” “work/employment troubles unrelated to interpersonal relationships,” and “other events such as disasters, incidents, accidents, or illness” (Table 7). The high‐neuroticism group reported a similar pattern (Table 8). The results for ACEs items are shown in Tables 9 and 10. Around 20% of participants endorsed each of the 10 ACEs items (range: 16.7%–29.5%). The most frequently reported included living with a household member with a mental illness (23.4%), domestic violence between parents or adults in the household (26.1%), psychological abuse (33.2%), physical abuse (29.5%), and psychological neglect (27.7%). The high‐HSP group reported significantly higher rates of parental mental illness, domestic violence, psychological abuse, physical abuse, and psychological neglect compared with the medium and low‐HSP groups (Table 9). The high‐neuroticism group had significantly higher rates of psychological abuse and psychological neglect compared with the other two neuroticism groups. Interestingly, the medium neuroticism group reported significantly higher rates of physical neglect, parental separation/divorce, household mental disorders, household substance abuse, household physical violence, incarceration of a household member, and sexual abuse compared with the other groups (Table 10).

**Table 9:** Adverse childhood experiences (ACEs) by HSP group (*n* = 2593).

Category	Total	%	Low‐HSP *n* = 354 (13.7%)			Medium‐HSP *n* = 1876 (72.3%)			High‐HSP *n* = 363 (14.0%)			*p*	*V*
			*n*	%	AR	*n*	%	AR	*n*	%	AR		
I was not given enough food, my clothes were dirty, or I felt that there was no one to protect or care for me.	630	24.3	83	23.4	−0.4	443	23.6	−1.3	104	28.7	2.1	0.118	0.041
I lost a parent due to divorce, neglect, or death.	611	23.6	81	22.9	−0.3	435	23.2	−0.7	95	26.2	1.3	0.447	0.025
I lived with someone who had depression, a mental illness, or had attempted suicide.	606	23.4	71	20.1	−1.6	427	22.8	−1.2	108	29.8	3.1	<0.01	0.065
I lived with someone suffering from alcohol or drug addiction (including prescription drugs).	522	20.1	72	20.3	0.1	362	19.3	−1.7	88	24.2	2.1	0.098	0.042
An adult in the household pushed, hit, slapped, harmed, or threatened another adult.	677	26.1	72	20.3	−2.7	478	25.5	−1.2	127	35.0	4.2	<0.001	0.091
I lived with someone who was incarcerated or sentenced to prison.	432	16.7	72	20.3	2	306	16.3	−0.8	54	14.9	−1	0.108	0.041
An adult in the household verbally abused, insulted, or belittled me.	860	33.2	80	22.6	−4.5	594	31.7	−2.6	186	51.2	7.9	<0.001	0.168
An adult in the household physically abused me by hitting, striking, or kicking me.	764	29.5	87	24.6	−2.2	531	28.3	−2.1	146	40.2	4.8	<0.01	0.099
I felt that no family member loved me or considered me special.	718	27.7	74	20.9	−3.1	495	26.4	−2.4	149	41.0	6.1	<0.001	0.127
I was forced to engage in unwanted sexual contact (such as touching, or oral, anal, or vaginal penetration).	450	17.4	59	16.7	−0.4	313	16.7	−1.5	78	21.5	2.2	0.081	0.044

*Note*: The percentage for Total represents the proportion relative to the total number of people, while the percentage for each group represents the proportion relative to the number of people in that specific group.

Abbreviations: AR, adjusted residual; HSP, highly sensitive personality.

**Table 10:** Adverse childhood experiences (ACEs) by neuroticism group (*n* = 2593).

Category	Total	%	Low‐neuroticism *n* = 286 (11.0%)			Medium‐neuroticism *n* = 1977 (76.2%)			High‐neuroticism *n* = 330 (12.7%)			*p*	*V*
			*n*	%	AR	*n*	%	AR	*n*	%	AR		
I was not given enough food, my clothes were dirty, or I felt that there was no one to protect or care for me.	630	24.3	42	14.7	−4	530	26.8	5.3	58	17.6	−3	<0.001	0.106
I lost a parent due to divorce, neglect, or death.	611	23.6	43	15.0	−3.6	506	25.6	4.4	62	18.8	−2.2	<0.001	0.088
I lived with someone who had depression, a mental illness, or had attempted suicide.	606	23.4	37	12.9	−4.4	504	25.5	4.6	65	19.7	−1.7	<0.001	0.098
I lived with someone suffering from alcohol or drug addiction (including prescription drugs).	522	20.1	30	10.5	−4.3	453	22.9	6.3	39	11.8	−4	<0.001	0.125
An adult in the household pushed, hit, slapped, harmed, or threatened another adult.	677	26.1	41	14.3	−4.8	542	27.4	2.7	94	28.5	1.1	<0.001	0.095
I lived with someone who was incarcerated or sentenced to prison.	432	16.7	26	9.1	−3.6	389	19.7	7.4	17	5.2	−6	<0.001	0.147
An adult in the household verbally abused, insulted, or belittled me.	860	33.2	61	21.3	−4.5	654	33.1	−0.2	145	43.9	4.4	<0.001	0.117
An adult in the household physically abused me by hitting, striking, or kicking me.	764	29.5	62	21.7	−3.1	591	29.9	0.9	111	33.6	1.8	<0.01	0.066
I felt that no family member loved me or considered me special.	718	27.7	43	15.0	−5.1	557	28.2	1	118	35.8	3.5	<0.001	0.114
I was forced to engage in unwanted sexual contact (such as touching, or oral, anal, or vaginal penetration).	450	17.4	30	10.5	−3.2	381	19.3	4.6	39	11.8	−2.8	<0.001	0.091

*Note*: The percentage for total represents the proportion relative to the total number of people, while the percentage for each group represents the proportion relative to the number of people in that specific group.

Abbreviation: AR, adjusted residual.

The results for perceptions of HSP and developmental disorders are presented in Tables 11 and 12. Among the participants, 966 (37.3%) reported being aware of HSP, and 1699 (65.5%) were aware of developmental disorders. In terms of self‐identification, 661 (25.5%) believed they were HSP, and 547 (21.1%) believed they had a developmental disorder. Regarding feelings about being labeled by others, 1004 (38.7%) expressed negative feelings about being called HSP, and 1393 (53.7%) expressed negative feelings about being labeled as having a developmental disorder. Regarding disclosure, 783 (30.2%) stated they would disclose being HSP, while 706 (27.2%) would disclose having a developmental disorder. Regarding identity, 772 (29.8%) considered being HSP part of their identity, while 691 (26.6%) considered having a developmental disorder part of their identity. The high‐HSP group was significantly more likely than the other HSP groups to answer “yes” to questions about awareness of HSP and developmental disorders, self‐identification with HSP or a developmental disorder, discomfort with being labeled as having a developmental disorder, willingness to disclose HSP or developmental disorder status, and considering either as part of their identity. There was no significant difference among HSP groups for discomfort with being labeled HSP (Table 11).

**Table 11:** Recognition of highly sensitive personality (HSP) and neurodevelopmental disorders by HSP group (*n* = 2593).

Category	Total	%	Low‐HSP *n* = 354 (13.7%)			Medium‐HSP *n* = 1876 (72.3%)			High‐HSP *n* = 363 (14.0%)			*p*	*V*
			*n*	%	AR	*n*	%	AR	*n*	%	AR		
You know about HSP.	966	37.3	106	29.9	−3.1	620	33.0	−7.2	240	66.1	12.3	<0.001	0.242
You know about developmental disorders.	1699	65.5	199	56.2	−4	1198	63.9	−2.9	302	83.2	7.6	<0.001	0.16
You think you are the HSP.	661	25.5	55	15.5	−4.6	386	20.6	−9.3	220	60.6	16.6	<0.001	0.327
You think you have a developmental disorder.	547	21.1	47	13.3	−3.9	341	18.2	−5.9	159	43.8	11.4	<0.001	0.228
You feel uncomfortable if others tell you that you are an HSP.	1004	38.7	135	38.1	−0.2	744	39.7	1.6	125	34.4	−1.8	0.169	0.037
You feel uncomfortable if others tell you that you have a developmental disorder.	1393	53.7	149	42.1	−4.7	1027	54.7	1.7	217	59.8	2.5	<0.001	0.099
If you were an HSP, you could tell people around you.	783	30.2	91	25.7	−2	533	28.4	−3.2	159	43.8	6.1	<0.001	0.121
If you had a developmental disorder, you could tell people around you.	706	27.2	97	27.4	0.1	480	25.6	−3	129	35.5	3.8	<0.001	0.077
Being an HSP is part of your identity.	772	29.8	92	26.0	−1.7	522	27.8	−3.5	158	43.5	6.2	<0.001	0.122
Having a developmental disorder is part of your identity.	691	26.6	89	25.1	−0.7	467	24.9	−3.3	135	37.2	4.9	<0.001	0.096

*Note*: The percentage for total represents the proportion relative to the total number of people, while the percentage for each group represents the proportion relative to the number of people in that specific group.

Abbreviation: AR, adjusted residual.

**Table 12:** Recognition of highly sensitive personality (HSP) and neurodevelopmental disorders by neuroticism group (*n* = 2593).

Category	Total	%	Low‐neuroticism *n* = 286 (11.0%)			Medium‐neuroticism *n* = 1977 (76.2%)			High‐neuroticism *n* = 330 (12.7%)			*p*	*V*
			*n*	%	AR	*n*	%	AR	*n*	%	AR		
You know about HSP.	966	37.3	102	35.7	−0.6	706	35.7	−2.9	158	47.9	4.3	<0.001	0.084
You know about developmental disorders.	1699	65.5	207	72.4	2.6	1239	62.7	−5.5	253	76.7	4.6	<0.001	0.11
You think you are the HSP.	661	25.5	41	14.3	−4.6	476	24.1	−3	144	43.6	8.1	<0.001	0.173
You think you have a developmental disorder.	547	21.1	25	8.7	−5.4	392	19.8	−2.8	130	39.4	8.7	<0.001	0.191
You feel uncomfortable if others tell you that you are an HSP.	1004	38.7	129	45.1	2.4	760	38.4	−0.5	115	34.8	−1.5	<0.05	0.052
You feel uncomfortable if others tell you that you have a developmental disorder.	1393	53.7	165	57.7	1.4	1028	52.0	−3.2	200	60.6	2.7	<0.01	0.064
If you were an HSP, you could tell people around you.	783	30.2	90	31.5	0.5	587	29.7	−1	106	32.1	0.8	0.595	0.02
If you had a developmental disorder, you could tell people around you.	706	27.2	93	32.5	2.1	530	26.8	−0.9	83	25.2	−0.9	0.085	0.044
Being an HSP is part of your identity.	772	29.8	94	32.9	1.2	587	29.7	−0.2	91	27.6	−0.9	0.354	0.028
Having a developmental disorder is part of your identity.	691	26.6	83	29.0	1	518	26.2	−0.9	90	27.3	0.3	0.579	0.021

*Note*: The percentage for total represents the proportion relative to the total number of people, while the percentage for each group represents the proportion relative to the number of people in that specific group.

Abbreviation: AR, adjusted residual.

The high‐neuroticism group was significantly more likely than the other neuroticism groups to answer “yes” to questions about awareness of HSP and developmental disorders, self‐identification with HSP or a developmental disorder, and discomfort with being labeled as having a developmental disorder (Table 12).

## DISCUSSION

The results of this study indicate that both the high‐HSP group and the high‐neuroticism tendency group exhibited high levels of depressive and anxiety symptoms, as well as high developmental traits associated with ASD and ADHD, with both groups displaying similar characteristics. Regarding past traumatic stress, both groups showed high rates of abuse and neglect in the family, interpersonal issues in the family, and bullying at school, and both groups also exhibited elevated PTSD symptoms. Concerning ACEs, both groups had a high prevalence of psychological abuse and psychological neglect, and the high‐HSP group further showed a higher rate of household mental disorders, household physical violence, and physical abuse.

In terms of the recognition of HSP and developmental disabilities, both groups showed a high rate of awareness of HSP and developmental disabilities, and reported a higher tendency to believe they were HSP or had a developmental disability, with a similar tendency to feel uncomfortable when others labeled them with a developmental disorder. However, there was no significant difference between the groups regarding the discomfort felt when labeled as HSP. On the other hand, the high‐HSP group showed a higher rate of believing that being HSP or having a developmental disability was part of their identity.

Based on these findings, it seems feasible to approach the relationship between psychiatric conditions such as depression, anxiety disorders, ASD, ADHD, and trauma‐related stress by using the scientifically established concept of neuroticism instead of the scientifically unestablished concept of HSP. Furthermore, it was suggested that considering the recognition of personal identity, especially in the high‐HSP group, where a greater proportion identified HSP or developmental disabilities as part of their identity, is essential for clinical involvement and intervention.

In this study, the proportion of the high‐HSP group (HSP) was 14.0%, which is slightly lower than the 15%–20% range,^1^ but generally consistent with their findings. It was also similar to the 12.88% proportion of HSP,^42^ indicating the validity of the classification used. Additionally, the proportion of high‐neuroticism tendency was 12.4%, which is similar to the proportion of the high‐HSP group. This also aligns closely with the 15%–20% range.^1^ Although the proportion of high‐neuroticism tendency was slightly lower than that of the high‐HSP group, it was almost the same.

HSP is associated with depression and anxiety disorders,^7^ and high neuroticism is related to depression and anxiety disorders.^43^, ^44^ In this study, the high‐HSP group and high‐neuroticism tendency group had significantly higher PHQ‐9 and GAD‐7 scores compared to the other groups, exceeding the cutoff values, further demonstrating the strong relationship between HSP, neuroticism, and depression, anxiety disorders, and mental health.

There is a strong relationship between HSP, ASD, and ADHD.^14^, ^45^ Previous studies have reported that HSP is positively correlated with neuroticism and openness^6^; however, in the present study, higher HSP levels were associated with higher neuroticism and lower extraversion. Furthermore, as neuroticism increased, not only extraversion but also conscientiousness and openness tended to decrease, suggesting differences in the levels of HSP and neuroticism.

According to previous research, genetic and brain imaging studies have suggested that both HSP and neuroticism are positively correlated with tendencies toward ASD and ADHD.^46^ In the present study, both the high‐HSP group and the high‐neuroticism group showed higher tendencies toward ASD and ADHD compared to the other groups; however, only ADHD exceeded the cutoff value, whereas ASD did not. A novel finding of this study is that in both the high‐HSP and high‐neuroticism groups, PTSD symptoms (IES‐R) were significantly higher, exceeding the cutoff value. Moreover, both groups exhibited prolonged responses to past traumatic experiences, suggesting the existence of a common pattern between them. Research on ACEs has reported that individuals with a history of childhood abuse tend to have stronger neurotic tendencies.^47^ However, research on the relationship between ACEs and HSP or neuroticism is limited, and it remains unclear which specific experiences are related to these traits. In the present study, both the high‐HSP and high‐neuroticism groups reported higher levels of psychological abuse and psychological neglect.

Additionally, the high‐HSP group had significantly higher rates of physical abuse, parental mental illness, domestic violence, and psychological abuse and neglect compared to other groups. To compare the strength of the associations with ACEs between the two traits, we calculated Cramér's *V* as an effect size. The analysis showed that the effect sizes for the high‐HSP group were comparable to those for the high‐neuroticism group across several ACE categories (e.g., psychological abuse: HSP *V* = [0.168], neuroticism *V* = [0.117]; psychological neglect: HSP *V* = [0.127], neuroticism *V* = [0.114]). The high‐neuroticism tendency group had more instances of psychological abuse and neglect compared to the middle neuroticism group, but the association with other forms of abuse was relatively weaker in terms of effect size. This suggests a need for further research to better understand the relationship between ACEs and HSP or neuroticism.

In both the high‐HSP group and high‐neuroticism tendency group among the general population, the levels of post‐traumatic stress exceeded the cutoff, suggesting that both groups had high responses to their most stressful past events.

There has been no prior research examining the awareness of HSP, but in this study, the recognition of HSP was found to be 37.3%, which is lower than the recognition of developmental disabilities at 65.5%. In the comparison between the groups, the high‐HSP group and the high‐neuroticism tendency group showed significantly higher awareness of both HSP and developmental disabilities compared to other groups. Furthermore, the proportion of individuals who recognized themselves as being HSP or having a developmental disability was also significantly higher in the high‐HSP and high‐neuroticism tendency groups compared to other groups.

Individuals who identify as HSP tend to embrace it as part of their identity, seeking social position and forming communities.^23^ In this study, it was found that in Japan, individuals with higher HSP tendencies are more likely to perceive HSP as part of their identity and are less likely to have strong negative feelings about being perceived as HSP by others. Regarding developmental disabilities, while people tended to have negative impressions of being perceived as having a developmental disability, they also recognized it as part of their identity. It was suggested that people with high HSP are more likely to identify their traits as part of their identity and feel comfortable expressing them to others. In contrast, although individuals with high neuroticism were also more likely to recognize themselves as having HSP or a developmental disability compared to other groups, they did not show a significant difference in recognizing these traits as part of their identity. Furthermore, there was a possibility that they might have negative impressions when others perceive them as having a developmental disability.

In conclusion, despite the differences in childhood adversities and the recognition of HSP or developmental disabilities as part of one's identity, the findings indicate a considerable similarity between HSP and neuroticism tendencies.

This study has several limitations. First, it used a specific survey company, which introduces bias due to using registered monitors. Future research should utilize random sampling to reduce this bias. Second, this study relied solely on a questionnaire survey, meaning that psychiatric diagnoses were self‐reported. Future studies should include psychiatric diagnoses based on structured interviews. Third, this study was limited to individuals aged 20–40, so it does not account for other age groups. Future research should explore personality traits, depression and anxiety symptoms, developmental characteristics, and stress in adolescents (teenagers) or individuals aged 50 and older. Fourth, the study only included male and female response options. Future studies should offer more inclusive options, such as male, female, other, prefer not to answer, or free‐text options, to ensure participants can respond comfortably. It is also difficult to capture qualitative information in this format. Fifth, regarding the analytical strategy, this study employed a trichotomous classification based on the mean ± 1 SD to prioritize the identification of high‐risk groups and clinical threshold effects. However, this approach may simplify the complex, continuous nature of personality traits. Future research should incorporate machine learning techniques, such as decision tree analysis or cluster analysis, to explore non‐linear patterns and complex interactions between HSP, neuroticism, and mental health indicators without relying on fixed cutoffs. Sixth, while we used Cramér's *V* to compare the strength of associations between ACEs and personality traits, we did not perform multivariate analyses (e.g., regression models) to simultaneously control for the intercorrelation between HSP and neuroticism. Future studies should employ multivariate approaches to further clarify the unique contributions of each trait to ACEs.

## CONCLUSION

The study suggests that both individuals with high‐HSP traits and those with high‐neuroticism tendencies often experience mental health conditions such as depression, anxiety, ASD, ADHD, trauma‐related stress, and ACEs. However, while closely related, neuroticism and HSP are not synonymous. Individuals with high‐HSP traits are often more likely to have experienced past abuse. Despite the higher prevalence of trauma in both groups, individuals with high‐HSP traits, unlike those with high neuroticism, may view HSP or developmental disorders as part of their identity. This recognition may empower them to share their experiences and connect with others in society.

## AUTHOR CONTRIBUTIONS

*Conceptualization*: all authors. *Project administration*: Eiji Shimizu. *Supervision*: Eiji Shimizu. *Statistical analysis*: Akari Matsuzawa. *Writing*—*original draft*: Akari Matsuzawa. *Writing*—*review and editing*: Eiji Shimizu, Akari Matsuzawa, and Tsubasa Sasaki.

## CONFLICT OF INTEREST STATEMENT

The authors declare no conflicts of interest.

## ETHICS APPROVAL STATEMENT

This study was approved by the Ethics Committee of Chiba University, Graduate School of Medicine (M10716).

## PATIENT CONSENT STATEMENT

Informed consents were obtained from all participants before participating.

## CLINICAL TRIAL REGISTRATION

Not applicable.

## Data Availability

The data are not publicly available and are not available for secondary use due to ethical considerations and participant confidentiality.

## References

[1] Aron EN , Aron A . Sensory‐processing sensitivity and its relation to introversion and emotionality. J Pers Soc Psychol. 1997 Aug;73(2):345–368. 9248053 10.1037//0022-3514.73.2.345

[2] Aron EN , Aron A , Jagiellowicz J . Sensory processing sensitivity: a review in the light of the evolution of biological responsivity. Pers Soc Psychol Rev. 2012 Aug;16(3):262–282. 22291044 10.1177/1088868311434213

[3] Pluess M . Individual differences in environmental sensitivity. Child Dev Perspect. 2015;9(3):138–143.

[4] Assary E , Zavos HMS , Krapohl E , Keers R , Pluess M . Genetic architecture of environmental sensitivity reflects multiple heritable components: a twin study with adolescents. Mol Psychiatry. 2021 Sep;26(9):4896–4904. 32488124 10.1038/s41380-020-0783-8 PMC8589650

[5] Smolewska KA , McCabe SB , Woody EZ . A psychometric evaluation of the highly sensitive person scale: the components of sensory‐processing sensitivity and their relation to the BIS/BAS and “Big Five”. Pers Individ Dif. 2006 Apr 1;40(6):1269–1279.

[6] Lionetti F , Aron EN , Aron A , Klein DN , Pluess M . Observer‐rated environmental sensitivity moderates children's response to parenting quality in early childhood. Dev Psychol. 2019 Nov;55(11):2389–2402. 31414847 10.1037/dev0000795 PMC7565718

[7] Dosari M , AlDayel SK , Alduraibi KM , AlTurki AA , Aljehaiman F , Alamri S , et al. Prevalence of highly sensitive personality and its relationship with depression, and anxiety in the Saudi general population. Cureus. 2023 Dec;15(12):e49834. 38164317 10.7759/cureus.49834 PMC10758235

[8] Jeronimus BF , Kotov R , Riese H , Ormel J . Neuroticism's prospective association with mental disorders halves after adjustment for baseline symptoms and psychiatric history, but the adjusted association hardly decays with time: a meta‐analysis on 59 longitudinal/prospective studies with 443 313 participants. Psychol Med. 2016 Oct;46(14):2883–2906. 27523506 10.1017/S0033291716001653

[9] Lüdtke O , Trautwein U , Husemann N . Goal and personality trait development in a transitional period: assessing change and stability in personality development. Pers Soc Psychol Bull. 2009 Apr;35(4):428–441. 19144768 10.1177/0146167208329215

[10] Ormel J , Oldehinkel AJ , Brilman EI . The interplay and etiological continuity of neuroticism, difficulties, and life events in the etiology of major and subsyndromal, first and recurrent depressive episodes in later life. Am J Psychiatry. 2001;158(6):885–891. 11384895 10.1176/appi.ajp.158.6.885

[11] Specht J , Egloff B , Schmukle SC . Stability and change of personality across the life course: the impact of age and major life events on mean‐level and rank‐order stability of the Big Five. J Pers Soc Psychol. 2011 Oct;101(4):862–882. 21859226 10.1037/a0024950

[12] Scher CD , Stein MB , Asmundson GJG , McCreary DR , Forde DR . The childhood trauma questionnaire in a community sample: psychometric properties and normative data. J Trauma Stress. 2001 Oct;14(4):843–857. 11776429 10.1023/A:1013058625719

[13] Bouchard TJ , McGue M . Genetic and environmental influences on human psychological differences. J Neurobiol. 2003 Jan;54(1):4–45. 12486697 10.1002/neu.10160

[14] Liss M , Mailloux J , Erchull MJ . The relationships between sensory processing sensitivity, alexithymia, autism, depression, and anxiety. Pers Individ Dif. 2008 Aug 1;45(3):255–259.

[15] Wakabayashi A , Baron‐Cohen S , Wheelwright S . Are autistic traits an independent personality dimension? A study of the Autism‐Spectrum Quotient (AQ) and the NEO‐PI‐R. Pers Individ Dif. 2006 Oct;41(5):873–883.

[16] Knouse LE , Traeger L , O'Cleirigh C , Safren SA . Adult attention deficit hyperactivity disorder symptoms and five‐factor model traits in a clinical sample: a structural equation modeling approach. J Nerv Ment Dis. 2013 Oct;201(10):848–854. 24080671 10.1097/NMD.0b013e3182a5bf33 PMC3821966

[17] Van Dijk FE , Mostert J , Glennon J , Onnink M , Dammers J , Vasquez AA , et al. Five factor model personality traits relate to adult attention‐deficit/hyperactivity disorder but not to their distinct neurocognitive profiles. Psychiatry Res. 2017 Dec;258:255–261. 28844557 10.1016/j.psychres.2017.08.037

[18] Gordon‐Lipkin E , Marvin AR , Law JK , Lipkin PH . Anxiety and mood disorder in children with autism spectrum disorder and ADHD. Pediatrics. 2018 Apr 1;141(4):e20171377. 29602900 10.1542/peds.2017-1377

[19] Suen YN , Chau APY , Wong SMY , Hui CLM , Chan SKW , Lee EHM , et al. Comorbidity of autism spectrum and attention deficit/hyperactivity disorder symptoms and their associations with 1‐year mental health outcomes in adolescents and young adults. Psychiatry Res. 2024 Jan;331:115657. 38056129 10.1016/j.psychres.2023.115657

[20] Grusnick JM , Garacci E , Eiler C , Williams JS , Egede LE . The association between adverse childhood experiences and personality, emotions and affect: does number and type of experiences matter? J Res Pers. 2020 Apr;85:103908. 32863469 10.1016/j.jrp.2019.103908 PMC7453784

[21] McLoyd VC . Socioeconomic disadvantage and child development. Am Psychol. 1998;53(2):185–204. 9491747 10.1037//0003-066x.53.2.185

[22] Luthar SS . Poverty and children's adjustment. Thousand Oaks (CA): Sage Publications, Inc; 1999. p. xii.

[23] Edenroth‐Cato F , Sjöblom B . Biosociality in online interactions: youths' positioning of the highly sensitive person category. YOUNG. 2022; Feb 1;30(1):80–96.

[24] Ioannou M , Olsson S , Bakken Wold A , Dellepiane M , Steingrímsson S . Approaching “highly sensitive person” as a cultural concept of distress: a case‐study using the cultural formulation interview in patients with bipolar disorder. Front Psychiatry. 2023 Sep 22;14:1148646. 37810603 10.3389/fpsyt.2023.1148646 PMC10558047

[25] Lewis‐Fernández R , Aggarwal NK , Bäärnhielm S , Rohlof H , Kirmayer LJ , Weiss MG , et al. Culture and psychiatric evaluation: operationalizing cultural formulation for DSM‐5. Psychiatry Interpers Biol Process. 2014;77(2):130–154. 10.1521/psyc.2014.77.2.130 PMC4331051 24865197

[26] Takahashi A . Development of Japanese version of the 19‐item Highly Sensitive Person Scale (HSPS‐J19). Jpn J Re Emot. 2016;23(2):68–77 (in Japanese).

[27] Pluess M , Assary E , Lionetti F , Lester KJ , Krapohl E , Aron EN , et al. Environmental sensitivity in children: development of the Highly Sensitive Child Scale and identification of sensitivity groups. Dev Psychol. 2018 Jan;54(1):51–70. 28933890 10.1037/dev0000406

[28] Lionetti F , Aron A , Aron EN , Burns GL , Jagiellowicz J , Pluess M . Dandelions, tulips and orchids: evidence for the existence of low‐sensitive, medium‐sensitive and high‐sensitive individuals. Transl Psychiatry. 2018 Jan;8(1):24. 29353876 10.1038/s41398-017-0090-6 PMC5802697

[29] Oshio S , Abe S , Cutrone P . Development of the Japanese version of the Ten Item Personality Inventory (TIPI‐J). Jpn J Pers. 2012;21(1):40–52 (in Japanese).

[30] Muramatsu K , Miyaoka H , Kamijima K , Muramatsu Y , Tanaka Y , Hosaka M , et al. Performance of the Japanese version of the Patient Health Questionnaire‐9 (J‐PHQ‐9) for depression in primary care. Gen Hosp Psychistry. 2018;52:64–69. 10.1016/j.genhosppsych.2018.03.007 29698880

[31] Muramatsu K , Miyaoka H , Kamijima K , Muramatsu Y , Tanaka Y , Hosaka M , et al. Validity and utility of the Japanese version of the GAD‐7: a study from the 51st Annual Meeting of the Japanese Society of Psychosomatic Medicine. Jpn J Psychosom Med. 2010;50(6):592 (in Japanese).

[32] Ustun B , Adler LA , Rudin C , Faraone SV , Spencer TJ , Berglund P , et al. The World Health Organization adult attention‐deficit/hyperactivity disorder self‐report screening scale for DSM‐5. JAMA Psychiatry. 2017 May;74(5):520–527. 28384801 10.1001/jamapsychiatry.2017.0298 PMC5470397

[33] Kessler RC , Adler L , Ames M , Demler O , Faraone S , Hiripi E , et al. The World Health Organization adult ADHD self‐report scale (ASRS): a short screening scale for use in the general population. Psychol Med. 2005 Feb;35(2):245–256. 15841682 10.1017/s0033291704002892

[34] Wakabayashi A , Tojo Y , Baron‐Cohen S , Wheelwright S . The Autism‐Spectrum Quotient (AQ) Japanese versions: evidence from high‐functioning clinical group and normal adults. Jpn J Psychol. 2004;75(1):78–84 (in Japanese). 10.4992/jjpsy.75.78 15724518

[35] Baron‐Cohen S , Wheelwright S , Skinner R , Martin J , Clubley E . The autism‐spectrum quotient (AQ): evidence from Asperger syndrome/high‐functioning autism, males and females, scientists and mathematicians. J Autism Dev Disord. 2001 Feb;31(1):5–17. 11439754 10.1023/a:1005653411471

[36] Asukai N , Kato H , Kawamura N , Kim Y , Yamamoto K , Kishimoto J , et al. Reliability and validity of the Japanese‐language version of the Impact of Event Scale‐Revised (IES‐R‐J): four studies of different traumatic events. J Nerv Ment Dis. 2002 Mar;190(3):175–182. 11923652 10.1097/00005053-200203000-00006

[37] Weiss DS . The Impact of Event Scale–Revised. In: Wilson JP , Keane TM , editors. Assessing psychological trauma and PTSD. 2nd ed. New York: Guilford Press; 2004. p. 168–189.

[38] Malinauskienė V , Bernotaitė L . The Impact of Event Scale ‐ Revised: psychometric properties of the Lithuanian version in a sample of employees exposed to workplace bullying. Acta Med Litu. 2016;23(3):185–192. 28356808 10.6001/actamedica.v23i3.3384 PMC5287992

[39] Kimura A , Hashimoto T , Niitsu T , Iyo M . Presence of psychological distress symptoms associated with onset‐related life events in patients with treatment‐refractory depression. J Affect Disord. 2015 Apr;175:303–309. 25661396 10.1016/j.jad.2015.01.027

[40] Oláh B , Fekete Z , Kuritárné Szabó I , Kovács‐Tóth B . Validity and reliability of the 10‐Item Adverse Childhood Experiences Questionnaire (ACE‐10) among adolescents in the child welfare system. Front Public Health. 2023;11:1258798. 38045975 10.3389/fpubh.2023.1258798 PMC10691263

[41] Felitti VJ , Anda RF , Nordenberg D , Williamson DF , Spitz AM , Edwards V , et al. Relationship of childhood abuse and household dysfunction to many of the leading causes of death in adults. The Adverse Childhood Experiences (ACE) study. Am J Prev Med. 1998 May;14(4):245–258. 9635069 10.1016/s0749-3797(98)00017-8

[42] Ueno Y , Takahashi A , Oshio A . Relationship between sensory‐processing sensitivity and age in a large cross‐sectional Japanese sample. Heliyon. 2019 Oct 3;5(10):e02508. 31667376 10.1016/j.heliyon.2019.e02508 PMC6812185

[43] Lahey BB . Public health significance of neuroticism. Am Psychol. 2009;64(4):241–256. 19449983 10.1037/a0015309 PMC2792076

[44] Kendler KS , Gatz M , Gardner CO , Pedersen NL . Personality and major depression: a Swedish longitudinal, population‐based twin study. Arch Gen Psychiatry. 2006 Oct;63(10):1113–1120. 17015813 10.1001/archpsyc.63.10.1113

[45] Panagiotidi M , Overton PG , Stafford T . The relationship between sensory processing sensitivity and attention deficit hyperactivity disorder traits: a spectrum approach. Psychiatry Res. 2020 Nov;293:113477. 33198048 10.1016/j.psychres.2020.113477

[46] Park SH , Guastella AJ , Lynskey M , Agrawal A , Constantino JN , Medland SE , et al. Neuroticism and the overlap between autistic and ADHD traits: findings from a population sample of young adult Australian twins. Twin Res Hum Genet. 2017 Aug;20(4):319–329. 28724480 10.1017/thg.2017.38

[47] Li ET , Luyten P , Midgley N . Psychological mediators of the association between childhood emotional abuse and depression: a systematic review. Front Psychiatry. 2020;11:559213. 33343409 10.3389/fpsyt.2020.559213 PMC7746653

